# Antisense Oligonucleotides Modulating Activation of a Nonsense-Mediated RNA Decay Switch Exon in the *ATM* Gene

**DOI:** 10.1089/nat.2016.0635

**Published:** 2016-12-01

**Authors:** Jana Kralovicova, Pedro M.D. Moreno, Nicholas C.P. Cross, Ana Paula Pêgo, Igor Vorechovsky

**Affiliations:** ^1^ Faculty of Medicine, University of Southampton, Southampton, United Kingdom.; ^2^ Instituto de Investigação e Inovação em Saúde (i3S), Universidade do Porto, Porto, Portugal.; ^3^ Instituto de Engenharia Biomédica (INEB), Universidade do Porto, Porto, Portugal.; ^4^ Wessex Regional Genetics Laboratory, Salisbury Hospital, Salisbury, United Kingdom.; ^5^ Faculdade de Engenharia and Instituto de Ciências Biomédicas Abel Salazar, Universidade do Porto, Porto, Portugal.

**Keywords:** alternative splicing, transposon, nonsense-mediated RNA decay, antisense oligonucleotides, *ATM*, lymphoid cancer, nanoparticles

## Abstract

*ATM* (ataxia-telangiectasia, mutated) is an important cancer susceptibility gene that encodes a key apical kinase in the DNA damage response pathway. *ATM* mutations in the germ line result in ataxia-telangiectasia (A-T), a rare genetic syndrome associated with hypersensitivity to double-strand DNA breaks and predisposition to lymphoid malignancies. ATM expression is limited by a tightly regulated nonsense-mediated RNA decay (NMD) switch exon (termed NSE) located in intron 28. In this study, we identify antisense oligonucleotides that modulate NSE inclusion in mature transcripts by systematically targeting the entire 3.1-kb-long intron. Their identification was assisted by a segmental deletion analysis of transposed elements, revealing NSE repression upon removal of a distant antisense *Alu* and NSE activation upon elimination of a long terminal repeat transposon MER51A. Efficient NSE repression was achieved by delivering optimized splice-switching oligonucleotides to embryonic and lymphoblastoid cells using chitosan-based nanoparticles. Together, these results provide a basis for possible sequence-specific radiosensitization of cancer cells, highlight the power of intronic antisense oligonucleotides to modify gene expression, and demonstrate transposon-mediated regulation of NSEs.

## Introduction

Eukaryotic genes contain intervening sequences or introns that must be removed by a large and highly dynamic RNA protein complex termed the spliceosome to ensure accurate protein synthesis [1]. The cell requires excessive energy and time to complete transcription of intron-containing precursor messenger RNAs (pre-mRNAs) from at least a quarter of the human genome and also needs to synthesize noncoding RNAs and >200 different spliceosomal proteins to achieve this task [1]. Although once regarded a selfish or junk DNA, introns are now recognized as critical functional components of eukaryotic genes that enhance gene expression and regulate alternative RNA processing, mRNA export, and RNA surveillance [2,3]. They are also an important source of new gene-coding and regulatory sequences [1,4,5] and noncoding RNAs, including microRNAs and circular RNAs [6,7]. Their removal process is tightly coupled with transcription, mRNA export, and translation, with most human introns eliminated from pre-mRNA cotranscriptionally [8]. However, their potential as targets for nucleic acid therapy is only beginning to be unleashed.

Spliceosomes assemble *ad hoc* on each intron in an ordered manner, starting with recognition of the 5′ splice site (5′ss) by the U1 small nuclear ribonucleoprotein or the 3′ss by the U2 pathway [1,9]. In addition to traditional splice site recognition sequences (5′ss, branch point, polypyrimidine tract, and 3′ss), accurate splicing requires auxiliary sequences or structures that activate or repress splice sites, known as intronic or exonic splicing enhancers or silencers. These elements allow genuine splice sites to be recognized among a vast excess of cryptic or pseudosites in vertebrate genomes that have similar sequences, but outnumber authentic sites by an order of magnitude [10]. Activation of cryptic splice sites can introduce premature termination codons (PTCs) in translational reading frames and may lead to genetic disease [11]. Such transcripts are usually recognized by a nonsense-mediated RNA decay (NMD) pathway and downregulated [12]; however, cryptic exons and NMD play also an important role in controlling the expression of naturally occurring transcripts [13] and differentiation stage-specific splicing switches, as exemplified by terminal stages of hematopoiesis [14,15]. In addition, cryptic splice sites may permit unproductive or partial spliceosome assemblies that may compete with natural splice sites, which may facilitate their accurate selection at a single-nucleotide resolution [16,17]. Cryptic splice sites can activate pseudoexons that limit gene expression (also known as poison or NMD switch exons), thus regulating the pool of mRNA isoforms and providing interesting targets for nucleic acid therapeutics [18]. However, potential exploitation of such strategies is in its infancy.

Splice-switching oligonucleotides (SSOs) are antisense reagents that modulate intron splicing by binding splice site recognition or regulatory sequences and competing with *cis-*elements or *trans-*acting factors for their targets [19–21]. They have been shown to restore aberrant RNA processing, modify the relative abundance of existing mRNA isoforms, or produce novel splice variants that are not normally expressed by the cell [20]. Most SSOs employed in preclinical and clinical development have targeted exonic sequences [19–21]. Whereas most exonic SSOs designed to induce exon skipping usually have a desired effect, functional intronic SSOs are more difficult to identify, unless they block access to intronic cryptic splice sites activated by a disease-causing mutation. First, a large fraction of intronic sequences may not affect RNA processing at all, despite the wealth of intronic auxiliary splicing motifs in the human genome [22]. In addition, a search for functional intronic SSOs that produce desirable RNA processing outcomes is usually inefficient and costly and may fail completely. For example, most SSOs systematically covering exon 7 of the *SMN2* (survival of motor neuron 2) gene stimulated exon skipping, a prerequisite for antisense therapy of spinal muscular atrophy; however, ∼20% SSOs increased exon inclusion [23]. By contrast, stimulation of intron splicing was found only for ∼10% of SSOs targeting *INS* intron 1, while the majority failed to show this effect [24]. Third, introns are enriched for many repetitive elements that preclude the SSO use with endogenous targets. Identification of effective intronic SSOs may be facilitated by global pre-mRNA folding and ultraviolet cross-linking and immunoprecipitation studies that identify binding sites for components of the spliceosome [18,25] or the exon junction complex [26]. However, these binding sites may not reflect optimal antisense targets and their resolution may be insufficient. Thus, identification of functional intronic SSOs remains challenging.

Our RNA-Seq studies have recently revealed activation of an NMD switch exon (termed NSE) deep in *ATM* (ataxia-telangiectasia, mutated) intron 28 in cells depleted of each subunit of the auxiliary factor of U2 small nuclear RNP (U2AF) [18]. U2AF binds to polypyrimidine tracts coupled with highly conserved 3′ss AG dinucleotides at intron ends and this binding promotes U2 recruitment to the branch site and formation of lariat introns [27–29]. However, recent identification of a large number of exons that were activated in cells depleted of each U2AF subunit (U2AF35 and U2AF65) and exhibited a distinct 3′ss organization [30,31] suggested that a subset of both canonical and NSEs is repressed by U2AF, similar to exon-repressing and -activating activities found for a growing number of RNA-binding proteins [32,33]. The NSE levels were responsive to knockdown of additional splicing factors involved in 3′ss recognition and were influenced by two natural DNA variants, rs609261 and rs4988000, located in the NSE 3′ss and further downstream, respectively [18]. We have also identified SSOs that modulate NSE inclusion levels in the *ATM* mRNA by targeting NSE and its competing pseudoexon in the same intron [18]. The *ATM* NSE provides an interesting and promising target for anticancer therapy for several reasons: (1) the ATM kinase is activated in response to double-strand breaks, mobilizing an extensive signaling network with a broad range of targets and influencing cellular sensitivity to DNA-damaging agents [34]; (2) the U2AF-regulated exon usage in the ATM signaling pathway is centered on the MRN/ATM-CHEK2-CDC25 axis and preferentially involves transcripts implicated in cancer-associated gene fusions and chromosomal translocations [18]; and (3) *ATM* NSE activation limits expression of ATM protein in cells lacking each U2AF subunit [18]. However, optimal NSE SSOs have not been defined and their delivery to lymphoid cells has not been tested.

In the present study, we have systematically screened SSOs covering unique sequences in the entire intron 28 and identify additional SSOs that activate or repress NSE *in vitro.* In the same intron, we also identify transposed elements that influence NSE inclusion in mature transcripts. Finally, we show efficient NSE repression upon SSO delivery to embryonic and lymphoblastoid cell lines using chitosan-based nanoparticles.

## Materials and Methods

### Plasmid constructs

**FIG. 1.**
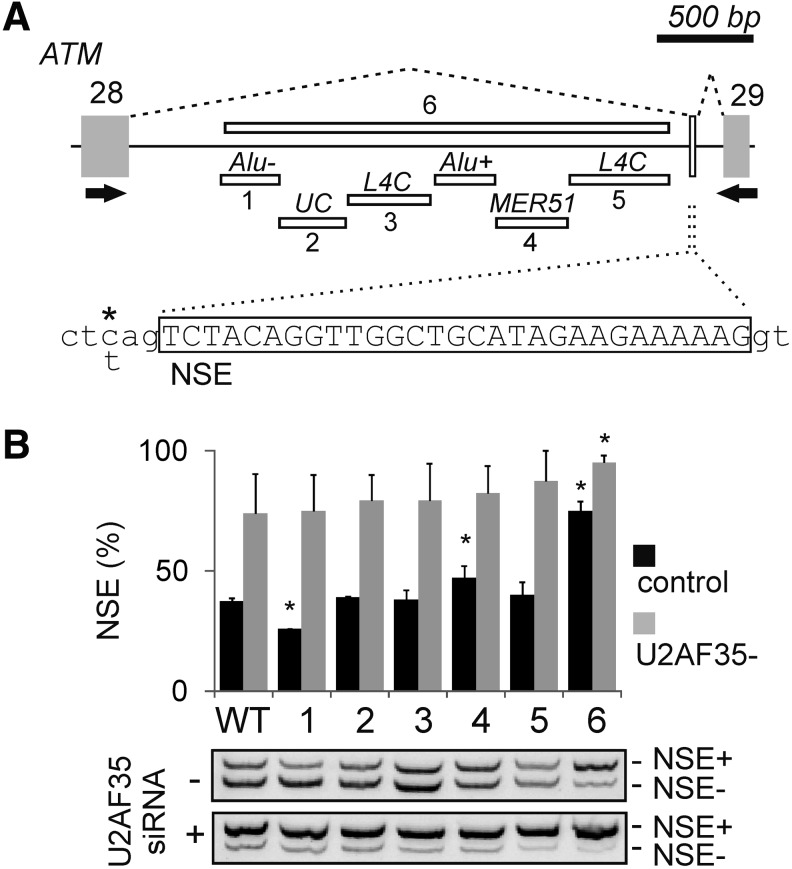
Identification of transposed elements in *ATM* intron 28 that influence NSE activation. **(A)** Location of transposed elements in intron 28 and schematics of NSE activation. Canonical exons [54] are shown as *gray boxes*, the NSE as a *white box*, introns flanking the NSE as *horizontal lines*, and their splicing by *dotted lines*. Deletions (numbered 1–6) of transposed elements are shown as *horizontal white rectangles*; UC, a unique sequence lacking recognizable transposons. Deletion numbers correspond to lanes in **(B)**. RT-PCR primers are denoted by *black arrows*. A scale is at the *top*. The NSE sequence is *boxed* in the *lower panel*. *Asterisk* denotes the C/T variant rs609261 located at the NSE 3′ss; rs4988000 (not shown) is 64 bp downstream of the NSE 5′ss. **(B)** Deletion of antisense *Alu* and MER51 alters NSE inclusion levels. WT and mutated constructs [designated 1–6 in **(A)**] were transiently transfected into HEK293 cells (mock) depleted of U2AF35. NSE+/−, RNA products with/without NSE. Columns represent mean NSE inclusion (%), error bars are SDs of two independent transfection experiments. *Asterisks* denote *P* values <0.01 for comparisons with the WT. 5′ss, 5′ splice site; *ATM*, ataxia-telangiectasia, mutated; NSE, NMD switch exon; RT-PCR, reverse transcription polymerase chain reaction; SDs, standard deviations; WT, wild-type.

Splicing reporter constructs containing full *ATM* intron 28 and flanking exons were obtained by ligating a ∼3.5 kb polymerase chain reaction (PCR) amplicon into the *Hin*dIII/*Xba*I site of pCR3.1 (Invitrogen). PCR was carried out with amplification primers, ATM26 and ATM27 (Table 1), and DNA from human embryonic kidney HEK293 cells as a template. PCR employed the Phusion^®^ High-Fidelity DNA Polymerase (Thermo Scientific) at the annealing temperature of 56°C and 1.5 mM MgCl_2_ for 30 cycles. Plasmids were propagated in *Escherichia coli* (strain DH5α). Plasmid DNA was extracted with the Gene JET Plasmid Miniprep kit (Thermo Scientific) and separated on 1.5% agarose gels to confirm the correct insert size following restriction enzyme digests prior to sequence validation. Deletion constructs (Fig. 1) were obtained by overlap extension PCR with mutagenic primers (Table 1) using the validated reporter plasmid with the full intron as a template. Each deletion insert was also fully sequenced to confirm the identity of intended changes and exclude undesired mutations. Hybrid *ATM* minigenes prepared by cloning ∼0.9-kb amplicons containing NSE and exon 29 into *Xho*I/*Xba*I sites of the *U2AF1* construct were described previously [18].

**Table 1.** Cloning, RT-PCR, and Mutagenic Primers

*Primer*	*5′-3′ Sequence*
Cloning primers	
ATM26	ataaagcttcttgttataaggttttgattcc
ATM27	atatctagatgtacataccctgaaaagtcac
RT-PCR primers	
PL4	agtcgaggctgatcagcgg
ATM-F	gagggtaccagagacagtgggatggc
ATM-R	ggctcatgtaacgtcatcaat
Mutagenic primers	
del-1F	atacaatttaccataatttacttttgaattatgtt
del-1R	aagtaaattatggtaaattgtatcatacattag
del-2F	ccttgccagaccagtttcctagttatctatattgaac
del-2R	taactaggaaactggtctggcaaggtggctta
del-3F	cttcaagggaccttggccgggtgcggtggct
del-3R	gcacccggccaaggtcccttgaagtttatctaa
del-4F	acacaaacaaagcttaggtttctttcttgtcaccttcta
del-4R	agaaagaaacctaagctttgtttgtgtgttttatacaa
del-5F	tgcctcatttacgtcatacaacttaatgatagacct
del-5R	ttaagttgtatgacgtaaatgaggcagggcaa
del-6F	tgatacaatttacctcatacaacttaatgatagacct
del-6R	attaagttgtatgaggtaaattgtatcatacattag

RT-PCR, reverse transcription polymerase chain reaction; restriction sites are *underlined*.

### Splice-switching oligonucleotides

**FIG. 2.**
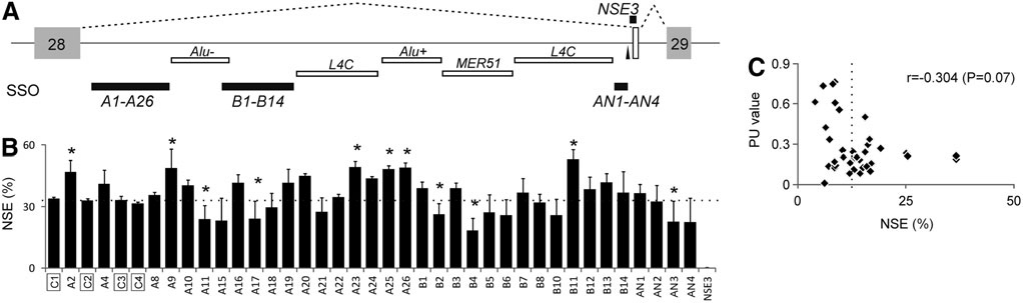
Identification of intronic SSOs that activate or repress NSE. **(A)** Location of tested SSOs in intron 28 relative to transposed elements (for legend, see Fig. 1A). The branch point sequence (GGCTGAT; branch point adenosine is *underlined*) of NSE is denoted by a *vertical arrowhead*. **(B)** Intronic SSOs that alter NSE inclusion in exogenous transcripts. SSOs are at the *bottom*. Multiple controls are *boxed*. SSO sequences are in Table 2. The average NSE inclusion in controls is denoted by a *dotted line*, error bars are SDs of two independent transfection experiments. *Columns* represent mean NSE inclusion levels, *asterisks* show significant *P* values. **(C)** SSOs targeting single-stranded regions tended to repress endogenous NSE. r, Pearson correlation coefficient. The *P* value is in *brackets*. SSOs, splice-switching oligonucleotides.

To test the effect of SSOs on both the endogenous and the exogenous *ATM* pre-mRNA, SSOs were designed to avoid transposed elements in intron 28. These elements were identified in the human reference sequence (hg19) with the crossmatch and slow options of the RepeatMasker web server (v.4.0.2; www.repeatmasker.org) [35] and confirmed in our constructs using the same method. The SSOs comprehensively covered three unique regions in *ATM* intron 28 (termed A, B, and AN, Fig. 2), avoiding only homopolymeric tracts. SSOs (Eurofins) were 2′-*O*-methyl modified at each ribose and by phosphorothioates at each end linkage to ensure adequate stability for the *ex vivo* screening. The GC content of SSOs was at least 24% (mean 31%) and their average length was ∼20 nt. SSOs were diluted in double-distilled water and quantified using Nanodrop (Thermo Scientific). Their normalized aliquots were stored at −80°C.

### Cell cultures and transfections of minigene constructs

HEK293 cells were maintained in standard culture conditions in Dulbecco's modified Eagle medium (DMEM) supplemented with 10% (v/v) of bovine calf serum. Cells were seeded at 50% confluency 24 h before transfections. Transfections of wild-type and deletion constructs were carried out in 12- or 24-well plates using jetPRIME (Polyplus) according to the manufacturer's recommendations, as described [30]. Briefly, 250 ng of plasmid DNA was mixed with 1.3 μL of jetPRIME in 75 μL of the jetPRIME buffer and incubated at room temperature for 15 min before adding into cell cultures. Each SSO was transfected with and without the full-length ATM construct using the same transfection reagent (2.5 μL) to a final concentration of 50 nM in each well. The cells were harvested 24 h later for total RNA extraction with TRI-Reagent (Ambion). Replicate experiments were carried out 1 week later.

### Determination of PU values

The PU (probability of *u*npaired) values estimate RNA single-strandedness using the equilibrium partition function by considering all possible RNA structures of short sequences, permitting their comparison at each nucleotide position [36]. Higher PU values indicate a higher single-strandedness of an RNA motif [36]. The PU values were computed as described [36] using the three unique intronic regions and their 30-bp flanks as an input. PU values for each position of an SSO target were averaged and the means were correlated with SSO-induced NSE inclusion levels.

### Preparation of stearylated trimethyl chitosan

Trimethyl chitosan, originally derived from ultrapure chitosan obtained from *Agaricus bisporus*, was provided by KitoZyme. Purified products had the number average molecular weight (*M_n_*) of 43.3 ± 5.5 kDa and the polydispersity index (*M_w_*/*M_n_*) of 2.4 ± 0.3, as determined by gel permeation chromatography in a 0.33 M NaCH_3_COOH/0.28 M CH_3_COOH eluent at a flow rate of 1 mL/min. The degrees of acetylation and quaternization, as determined by the Fourier-transform infrared spectroscopy and ^1^H-nuclear magnetic resonance spectroscopy (^1^H NMR) [37], respectively, were 11.1% ± 0.9% and 30.1% ± 4.6%. Trimethyl chitosan was functionalized with N-succinimidyl stearate (Santa Cruz Biotechnologies), as previously described [37], achieving a final degree of substitution of 2.1% ± 0.6% (mol %), as determined by ^1^H NMR.

### Formation and delivery of nanocomplexes

The nanocomplexes were prepared by mixing equal volumes (30 μL) of SSO and polymer solutions, as described previously [37]. Briefly, SSOs were diluted in buffer A [20 mM HEPES, pH 7.3, 5% (w/v) glucose] and supplemented with 1 M Na_2_SO_4_ to a final concentration of 50 mM. Both the polymer and SSO solutions were heated at 60°C for 5 min before mixing with vortex at 1,000 rpm for 15 s. The tested complexes were prepared with molar ratios of quaternized amines (N) to phosphate groups (P) of 20, 40, and 80 (N/P ratios), as previously optimized for the first SSOs [37]. Their hydrodynamic diameter was between 110 and 130 nm for the N/P ratios between 20 and 80. The complexes were allowed to stabilize at room temperature for 30 min before adding to 240 μL DMEM without serum and antibiotics. A final concentration of SSOs in chitosan-containing cultures was 300 nM. Twenty-four hours after addition of complexes, 300 μL of the culture medium with serum/antibiotics was added to HEK293 or VAVY cells. The cells were harvested 24 h later for total RNA extraction. Replicate experiments were carried out 1 week later.

### Analysis of spliced products and NSE measurements

Total RNA samples from chitosan experiments were extracted with the RNeasy kit (Qiagen). RNA was quantified using Nanodrop (Thermo Scientific) and 1 μg of total RNA was reverse transcribed with the Moloney murine leukemia virus reverse transcriptase (Promega) and oligo-d(T) primers. Exogenous complementary DNA (cDNA) samples were amplified using primers, PL4 and ATM-F, and endogenous products were amplified with primers, ATM-F and ATM-R (Table 1), and GoTaq polymerase (Promega) for 28 cycles (the annealing temperature was 56°C at 1.5 mM MgCl_2_). Spliced products were separated on 1.5% agarose and then on 6% polyacrylamide gels. The signal intensities were measured in polyacrylamide gels using FluorQuant and Phoretix software packages (Nonlinear Dynamics, Inc.) to obtain the percentage of NSE inclusion in polyadenylated RNAs.

### Branch site prediction

The NSE branch point was predicted using the SVM-BP finder (available at http://regulatorygenomics.upf.edu/Software/SVM_BP/). The SVM score of the indicated NSE branch site was 1.3.

### Statistical analysis

Descriptive statistics were computed with Stat200 (BioSoft). Mean NSE inclusion levels were compared using *t*-tests; the indicated *P* values are two-tailed.

## Results

SSOs targeting either 3′ or 5′ss of the *ATM* NSE efficiently repress this exon in a haplotype-dependent manner [18]. To facilitate identification of optimal intronic SSOs that increase NSE inclusion in mature transcripts, we first prepared splicing reporter constructs with the entire *ATM* intron 28 (Fig. 1A). The construct was obtained by PCR using the HEK293 DNA as a template. The reference sequence (hg19) of human intron 28 is ∼3.1 kb long, which is similar to the average human intron [38]. About 64% of this intron is occupied by transposed elements, filling completely its middle part, except for a ∼350 bp region in the 5′ half of the intron and exonic flanks (Fig. 1A). Plasmid DNA sequencing revealed the same organization of transposed elements without any additional transposon copies. It also showed the C and G alleles at rs4988000 and rs609261, respectively, indicating that the construct contained the haplotype most permissive for NSE inclusion in the *ATM* mRNA [18]. Following transfections into HEK293 cells, total RNA was extracted and reverse transcribed before amplification with a combination of a vector primer PL4 (Table 1) and an exon primer [18] (Fig. 1A). Examination of spliced products showed that most transcripts entirely lacked intronic sequences (NSE−), whereas ∼36% of the mRNA contained NSE (NSE+, Fig. 1B, lane 1). This fraction was slightly higher than for a hybrid *ATM* reporter examined previously [18], consistent with the presence of auxiliary splicing sequences in newly cloned intronic segments.

To determine the importance of transposed elements for NSE inclusion, we individually deleted each transposon from intron 28 (deletions 1–5, Fig. 1A). We also deleted a large middle part of the intron along with all transposons, leaving the NSE and its upstream sequences intact, including the predicted branch site (∼75% of the intron, deletion 6). Transfection of validated mutated constructs, which all had identical genotypes to the wild-type construct at rs4988000 and rs609261, revealed that the large deletion promoted NSE-containing transcripts (deletion 6, Fig. 1B). Deletion of the MER51 element increased NSE inclusion to a lesser extent. In contrast, deletion of the antisense *Alu* inhibited NSE, while deletion of long interspersed repeats (deletions 3 and 5) or a unique intronic segment (deletion 2) had no effect on NSE inclusion. The variability of NSE inclusion levels was higher following transfections of the same plasmids into cells depleted of U2AF35, with a significant increase of NSE levels maintained only for deletion 6 (Fig. 1B), consistent with a major stress component of NSE responses [18].

We next designed a series of SSOs targeting three intronic regions that have unique sequences in the genome (termed A, B, and AN) while avoiding a predicted branch site upstream of NSE (Fig. 2A and Table 2). Each SSO was modified with 2′-*O*-methyl at each ribose and phosphorothioate at each end linkage to ensure their RNase H resistance and sufficient stability in transient transfections. As a positive control, we used SSO-NSE3, which was highly efficient in blocking the NSE 3′ss [18]. As negative controls, we employed a series of scrambled SSOs and SSOs targeting other genes, including *INS* [24] and *BTK* [39] (Table 2), which were not expressed in HEK293 cells, as confirmed by our RNA-Seq data [30]. Each SSO was individually transfected with or without the wild-type *ATM* construct. Measurements of spliced products revealed that SSO-NSE3 yielded the most efficient NSE repression, as expected (Fig. 2B). About a half of tested SSOs significantly altered NSE inclusion levels compared with controls, with similar numbers of repressor and activator SSOs. Pearson correlation coefficient between replicate transfections was highly significant, reaching 0.88 (*P* < 10^−8^); however, the overall correlation between exogenous and endogenous NSE levels was only 0.35 (*P* < 0.01).

**Table 2.** Splice-Switching Oligoribonucleotides

*SSO*	*5′-3′ Sequence*^a^
A2	aacuuaaagguuauaucuc
A4	uauaaauacgaauaaaucga
A8	cauggguuggcuaugcuag
A9	caacacgacauaaccaaa
A10	aagccaaucagagggagaca
A11	aacauuucuauuuaguuaaaagc
A15	ucguguauuacaacaguuaa
A16	caaccaguuugcauucgu
A17	uuaguauuccuugacuuua
A18	uucuguacacuguuuaguauucc
A19	gaagagggagugaagguu
A20	aaagcuuggugagauuga
A21	uuucuugaaaaguggaaagcuug
A22	uggaaugagggacgguuguuuuuc
A23	gguaugagaacuauagga
A24	aaacaaacagcaggguau
A25	gguaauaagugucacaaa
A26	guaucauacauuagaagg
B1	ucaaaaguaaauuauggucu
B2	gacugguaaauaauaaacauaauuc
B3	aaauguauacuggagaagacu
B4	auauauuagagauacaucagcc
B5	gacaaacauuuaaugaauacucaa
B6	uugacuccuucuuuugacaaacau
B7	uuuaaauccuuccuuacuu
B8	gauuauaaaacaaacgaagc
B10	uguuuuaauauaaguugcuucaa
B11	uguggggugaccacagcuu
B12	ucccuuacuuauauccaa
B13	ccaaguuugguuacuuauc
B14	gaaguuuaucuaauauugacc
AN1	ggucuaucauuaaguuguauga
AN2	uuaaauaagacuucaggucua
AN3	uuagagaaucauuuuaaauaagac
AN4	cuuaauccaauucuucaauuuuag
C1	aggugcucgcgggugg
C2	guugcaaauuucuucaaauc
C3	agcuggggccuggggu
C4	ggaaacuugcccguguucca

^a^ Each SSO was modified with 2′-*O*-methyl at each ribose and phosphorothioate at each end linkage.

SSO, splice-switching oligonucleotide.

Experiments in Fig. 1 showed that NSE inclusion is controlled by distant splicing regulatory sequences within and outside transposons. Splicing enhancer and silencer motifs in their natural pre-mRNA context occur preferentially in single-stranded regions [36], suggesting that they are more accessible to RNA-binding proteins or other ligands that control exon selection. Preferential targeting of SSOs to unpaired regions could thus improve our search strategy. To test this assumption, we correlated endogenous NSE inclusion levels after the SSO treatment with their average PU values (Fig. 2C). These values estimate single-strandedness of their RNA targets using an equilibrium partition function, with higher values signaling a higher probability of single-stranded conformation [36]. Interestingly, SSO targets with higher average PU values tended to induce exon skipping, suggesting that efficient blocking of unpaired interactions as far as 2 kb from the exon can impair its inclusion levels in the mRNA.

The experiments described above identified a small set of intronic SSOs that increased NSE inclusion in exogenous and endogenous mRNAs and that might serve as gene-specific repressors since NSE can limit ATM expression through NMD [18]. ATM repression by NSE-activating SSOs might be advantageous for cancer treatment by inhibiting the double-strand break signaling pathway and radiosensitization [40]. To test if *ATM* SSOs can be delivered to cells that have much lower transfection efficiency than HEK293 cells, we employed a stearylated trimethylated chitosan (TMC-SA). Chitosan is a natural copolymer of d-glucosamine and *N*-acetyl-d-glucosamine known for biocompatibility, biodegradability, and low toxicity and immunogenicity [41,42]. When trimethylated, chitosan acquires a permanent positive charge that improves its solubility at neutral pH [41]. Stearylation was found to be necessary for formation of stable nanocomplexes with SSOs and their transfection activity [37] in the HeLa/pLuc705 system, which makes use of a luciferase gene interrupted by a mutated *HBB* intron [43].

**FIG. 3.**
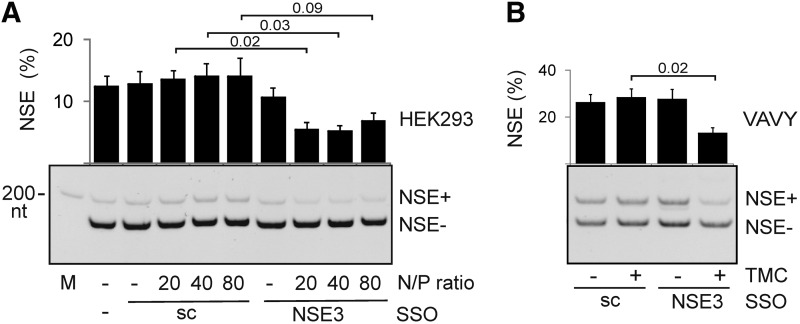
TMC-SA-assisted delivery of SSO-NSE3 to human cell lines leads to NSE repression. **(A)** NSE inclusion in HEK293 cells is inhibited upon exposure of SSO-NSE3/TMC-SA nanocomplexes. Sc, a scrambled control with the same modification. M, size marker. Error bars denote SDs of two transfection experiments. *P* values are shown at the *top* for the indicated comparisons. **(B)** NSE repression in VAVY cells exposed to the SSO-NSE3/TMC-SA nanocomplexes. TMC-SA, stearylated trimethylated chitosan.

We first tested if TMC-SA can facilitate delivery of SSO-NSE3 into HEK293 cells. Figure 3A shows reduction of NSE levels following exposure to the TMC-SA/SSO-NSE3 nanocomplexes compared with a complexed scrambled SSO. This decline was significant for the TMC-SA/SSO-NSE3 (N/P) ratios 20 and 40. NSE reduction was also apparent when comparing NSE inclusion in cells exposed to uncomplexed SSO-NSE3, consistent with their significant uptake by this highly transfectable cell line. However, the decline of NSE levels was smaller for TMC-SA/SSO-NSE3 than for the same oligo transfected with jetPrime to the same cell line at a lower final concentration. Finally, significant NSE repression upon exposure to the TMC-SA/SSO-NSE3 nanocomplexes was observed also for a lymphoblastoid cell line where uncomplexed SSO-NSE3 failed to reduce NSE altogether (Fig. 3B).

## Discussion

In this work, we have shown for the first time that a chitosan-based delivery system for intronic SSOs can repress an NSE (Fig. 3). Our results also demonstrate the first transposed elements that promote or repress inclusion of an NSE in mature transcripts (Fig. 1).

**FIG. 4.**
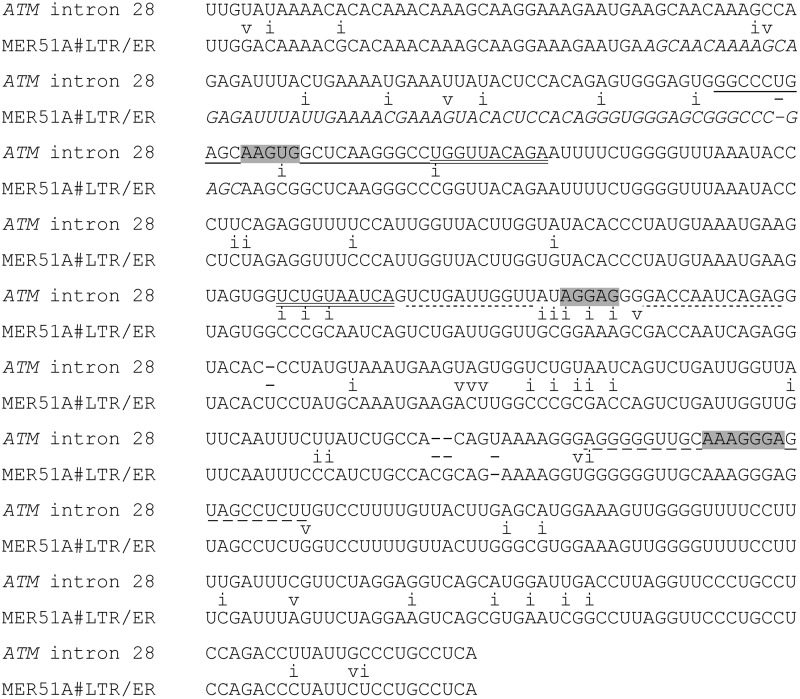
Inverted repeats in the MER51 element of *ATM* intron 28. The alignment was carried out by RepeatMasker [35]. v, transversions; i, transitions. Putative purine-rich loops (highlighted in *gray*) are flanked by inverted repeats (*underlined*) that may form stable base-pairing interactions during transcription. The long terminal repeat homology region originally described for the MER51 family [49] is in *italics*. The aligned segment corresponds to deletion 4 shown in Fig. 1A. The MER51A consensus sequence is in the antisense orientation.

*Alu* sequences have a propensity to exonize through 3′ss or 5′ss activation [4,44] or auxiliary splicing motifs [17,45] upon a single mutation. These events contribute significantly to human morbidity [46]. In addition, they can be exonized by outlying deletions and cause genetic disease [47], indicating that they can promote inclusion of remote intronic sequences in mature transcripts. Although the exact mechanism of such distant effects is not understood, the secondary structure of these GC-rich transcripts is likely to play a major role [46,48]. However, mutation-induced exonization has been shown for all other classes of transposed elements, including more ancient, short interspersed elements termed mammalian interspersed repeats [46]. In the present study, an intronic transposed element with the highest similarity to MER51A (MEdium Reiterated frequency repeat, family 51, [49]) repressed NSE, acting as a buffer to counteract the *Alu*-mediated NSE activation (Fig. 1A, B). The *ATM* MER51 is also relatively GC-rich (∼44%), which could facilitate intramolecular interactions with GC-rich *Alu*s during cotranscriptional folding. The element contains several inverted repeats that might form stable hairpins, exposing purine-rich loops (Fig. 4) that could act as splicing regulatory motifs. These stem-loops should be examined in future studies to identify distant interactions underlying the MER-mediated exonization. About 250,000 copies of recognizable MER sequences were estimated to exist in the human genome [38,50] and many were found in mature transcripts of protein-coding genes, contributing to the diversity of protein interactions [51]. A mutation-induced MER exonization was also shown to cause Gitelman syndrome [52]. The 3′ part of MER51 is similar to long terminal repeats of retroviruses (Fig. 4) [49], which account for ∼15% of transposon-mediated exonization events leading to human genetic disease [46]. The origin of most MERs was placed after the decline of mammalian interspersed repeats before the spread of *Alu*s, coinciding with the expansion of mammals and suggesting that MERs may offer insights into early mammalian radiation [49]. Taken together, our results suggest that the interplay of transposed elements in long introns could influence inclusion levels of many NSEs, fine-tuning gene expression.

We have also identified candidate sequence-specific ATM inhibitors that act by promoting a regulated NSE important for ATM expression (Fig. 2). ATM inhibitors sensitize cancer cells to cytotoxic therapy that induces double-strand breaks, including local radiotherapy, an integral part of treatment regimens of many cancer types [53]. Although chemical ATM inhibitors showed great promise, their undesired pharmacokinetic profiles, high toxicity, or poor efficacy has hampered their progression into the clinic [53]. In contrast, SSOs target unique sequences in the human genome, can be delivered using natural biodegradable compounds (Figs. 1–3), and their mechanism of action is better defined. In addition, the availability of NSE-activating and -repressing SSOs may offer an opportunity to titrate gene expression more accurately than less specific chemical inhibitors, assuming their efficient delivery to newly identified intron 28 targets. Although NSE is included in natural transcripts at low levels, it can be dramatically upregulated in response to various stimuli [18,54], requiring further studies into NSE regulation. Recently, a gene-specific antisense inhibition of NMD employed SSOs targeting exon junction complex deposition sites, thus permitting NMD repression without relying on skipping of a PTC-containing exon [26]. The two approaches, the former relying on intronic sequence and the latter on exonic targets, might complement each other in the future to expand the repertoire of antisense strategies that inhibit NMD.

The average length of SSOs employed in our screening was close to the minimum for unique targets (Table 2). Shorter SSOs may induce more off-target effects than longer SSOs, which could contribute to the observed low correlation between NSE inclusion levels in endogenous and exogenous transcripts. Apart from the possible suboptimal target specificity, intron 28 splicing and NSE inclusion can be influenced by adjacent introns that were absent in exogenous transcripts. In addition, intron 28 splicing may not be strictly cotranscriptional [18]. Furthermore, distinct promoters of exo- and endogenous transcripts could be associated with dissimilar RNA folding or its kinetics, further contributing to the low correlation. Nevertheless, our study clearly demonstrates a wealth of candidate intronic target sites for functional SSOs, in agreement with a high information content of human intronic auxiliary splicing sequences. This content is higher than in lower organisms, which have smaller introns with a lower regulatory potential for alternative splicing [22].

Although SSO-NSE3 and other SSOs can repress endogenous NSE-containing mRNAs (Fig. 2B, C) [18] and NMD transcripts with the relative abundance as low as ∼1% can contribute to the mRNA consumption [55], it remains to be tested if their reduction can lead to a sustained increase of ATM protein levels in normal cells. Unlike cells depleted of U2AF subunits where NSE inclusion is high (Fig. 1) and ATM signal on immunoblots was increased upon a single SSO transfection [18], the low endogenous NSE levels in normal cells would necessitate a prolonged and repeated SSO exposure and more sensitive assays, ideally coupled with functional analysis of ATM signaling targets. Newly identified SSOs might also alleviate phenotypic consequences of leaky ataxia-telangiectasia (A-T) alleles in a mutation-independent manner, especially in homozygous A-T patients carrying the C allele at rs609261, which facilitates 3′ss recognition of the NSE [18]. Since chitosan-based nanoparticles have been shown to penetrate the blood–brain barrier and accumulate in cerebellum [56], SSO-NSE3 and other NSE repressors might have a future therapeutic potential in slowing down the progression of cerebellar symptoms of a subset of A-T patients.
